# Impact of the Atomic Packing Density on the Properties of Nitrogen-Rich Calcium Silicate Oxynitride Glasses

**DOI:** 10.3390/ma15176054

**Published:** 2022-09-01

**Authors:** Sharafat Ali

**Affiliations:** Department of Built Environment and Energy Technology, School of Engineering, Linnæus University, SE-351 95 Vaxjo, Sweden; sharafat.ali@lnu.se

**Keywords:** oxynitride glass, atomic packing density, refractive index, hardness, high nitrogen content, high calcium content

## Abstract

In this work, the impact of the atomic packing density/fractional glass compactness of Ca–Si–O–N glasses on glass transition and crystallization temperatures, glass density, microhardness, molar volume, and refractive index were examined. It was found that the atomic packing density increased with increasing the nitrogen content and decreased with increasing the Ca content in the glass network. Furthermore, density, glass transition and crystallization temperatures, and refractive index, increased with an increasing atomic packing density of the glass, while molar volume increased with decreasing atomic packing density values. The change in hardness with atomic packing density is less clear and suggests that the atomic packing density does not solely control the underlying deformation mechanism. There is indeed competition between densification (favored at low packing density values) and isochoric shear (at larger packing density). Despite that, the effects of nitrogen as a network former and Ca as a modifier are significantly independent. The obtained results indicate that the atomic packing density of the prepared samples linearly depends on many mechanical and optical properties, suggesting that the glass network and cross-linking are proportional to the ionic radius of the Ca and the nitrogen content, respectively.

## 1. Introduction

It is well-known that the incorporation of nitrogen (*N*) into the oxide glass network exhibits superior mechanical properties such as toughness, elastic moduli, and microhardness; furthermore, the glass transition temperature ($T_{g}$), crystallization temperature ($T_{c}$), and refractive index (*RI*) also significantly increase with N content. These types of trends are most likely due to the partial replacement of two-fold coordinated oxygen atoms with three-fold coordinated N. Such observations have been confirmed using several techniques, including FTIR, X-ray photoelectron spectroscopy (XPS), and nuclear magnetic resonance (NMR) [1,2]. It is also important to mention that the incorporation of N in the glass generally provides a more tightly and highly linked glass network [3,4,5,6,7,8].

Oxynitride glasses are typically synthesized in a wide range of oxide systems, e.g., silicate, aluminosilicate, boroaluminosilicate, fluoroaluminosilicate, borate, borophosphate, phosphate, phosphosilicate, and phosphoaluminosilicate glasses. Silicon-based oxynitride glasses have been studied most, both from preparation and properties point of view, compared to other oxynitride glasses. These glasses can be prepared by melting mixtures containing glass modifier metal oxide, SiO_2_, and Si_3_N_4_/AlN, yielding glasses with N content up to typically c.a. 25 e/o. Recently, few studies have reported that AE/RE–Si–O–N (where “AE” is alkaline-earth and “RE” is rare-earth) glasses can be formed by incorporating a metal modifier such as metal hydride or even pure metal in the reaction mixture rather than using metal oxides, as typically used with silicon nitride (Si_3_N_4_) as the N source yielding oxynitride glasses, with N content of up to 60 e/o with exceptional thermal, mechanical, and optical properties [9,10,11,12,13,14,15,16].

For oxide glasses [17,18,19] as well as for oxynitride glasses [20,21,22,23,24], several studies have shown that many mechanical properties such as elastic properties, hardness, and thermal properties, e.g., glass transition temperature ($T_{g}$) and crystallization temperature ($T_{c}),$ depend on three main factors: (1) the type of *AE/RE* element, (2) the amount of the *AE/RE* in the glass system, and (3) the amount of N. [25,26]. Rouxel et al. [27] discussed the elastic properties of glasses in the light of their short to medium-range structural features (i.e., coordination number, cross-linking between rings, sheets, and chains, the dimensionality of the structural units, and interatomic bonding). It is noteworthy to mention that owing to the unknown of many structural properties such as bond energies, bond lengths, and bond angles, it is difficult to analyze the atomic structure distribution of the glass precisely. Interestingly, few properties, such as atomic packing density/fractional glass compactness, can be associated with the glass properties. The atomic packing density of glass is a measure of how well the ions are packed in the glass. Atomic packing density is not known with high accuracy because ionic radii depend on the fine details of the atomic organization (coordination, valency, affinity with the neighboring atoms). Furthermore, the meaning of an ionic radius is questionable as the interatomic bonds increase, for instance, when more covalent species such as N are introduced. Nevertheless, in general, atomic packing density provides some insight into the trends in properties [27]. In oxide glasses, where the atomic network is built on chiefly ionic bonds, cation field strength (*CFS*) is another useful parameter (for instance, to differentiate glasses which only differ by the nature of the rare-earth oxide introduced); however, the situation is more complicated in the presence of strong covalent bonds as in oxynitride glasses [7].

The addition of N and modifier to a series of Ca–Si–O–N glasses was investigated by Sharafat et al. [12,13,28] and unraveled the effect of N on glass properties. The present work discusses the relationships between the atomic packing density ($C_{g}$), molar volume (Mv), density (*ρ*), glass transition temperature $\left(T_{g}\right)$, Vickers hardness ($H_{v}$), and refractive index (*RI*) of Ca–Si–O–N glasses with varying calcium (Ca) and nitrogen (N) contents.

## 2. Experimental

In this study, a series of Ca–Si–O–N samples with a high amount of N and modifier were selected, as displayed in Table 1, and the specimen was labeled accordingly, increasing N content in the glass network. In addition, all of the compositions presented in this work are expressed as an equivalent percent [N content in equivalent; e/o = (3[N] × 100)/(2[O] + 3[N]), where [O] and [N] are the atomic concentrations of nitrogen and oxygen, and 3 and 2 are their respective valences]. Oxynitride glasses in the calcium silicon oxynitride system were fabricated by the traditional melt-quenching technique from mixtures of Calcium hydride (purity98%), purchased from Alfa Aesar GmbH & Co. (Kandel, Germany), and Si_3_N_4_ and SiO_2_ powders purchased from ChemPur GmbH (Karlsruhe, Germany) and ABCR GmbH & Co. (Karlsruhe, Germany), respectively. Subsequently, an equal volume mixture (6 gm batches) of each composition was mixed and ground inside a glove box filled with argon to avoid the oxidation of the mixture. After that, the powders were placed in Nb crucibles and heated up at 1500–1700 °C via a high-temperature furnace. More details on the fabrication and characterizations of the samples can be found in our previously published works [8,20]. The Archimedes method was used to measure the density of the bulk oxynitride glass samples. The weight of each prepared sample was measured five times in water and air to calculate the standard deviation in each measurement. The molar volume (Mv) of the glass sample consisting of *i* elements was calculated through the following formula:

(1) $$Mv=\frac{\sum_{i}x_{i}\;m_{i}}{\rho },$$

where X_i_, M_i,_ and ρ are the mole fraction, molar mass, and the density of the glass, respectively.

The atomic packing density $\left(C_{g}\right)$ of the glasses was obtained from the total theoretical volume of all ions in a mol of glass and the effective molar volume of the glass using the following formula:

(2) $$C_{g}=\;\frac{\sum_{i}X_{i}V_{i}\;N}{Mv},$$

where N is a constant called Avogadro’s number, while *V_i_* is the ion volume of the element “*i*”. *Vi*, the volume of the ionic species, can be calculated through the ionic radii (r) given by Shannon [29] using the equation.

(3) $$V_{i^{\;}}=\frac{4}{3}\pi_{i}r^{3}.$$

A Netzsch STA 409PC instrument was used to measure the thermal properties ($T_{g}$ and $T_{c}$) of the prepared samples. To measure the thermal properties, crushed pieces of prepared glass were placed in Pt cups and heated up to 1350 °C with a ramp rate of 20 °C/min in flowing nitrogen. A Matsuzawa microhardness tester (Model: MXT-α 1 with a pyramid-shaped diamond indenter) was used to measure the microhardness of the glasses at room temperature. Through a series of indentation experiments (10 indentation experiments for each sample) with an indentation load of 300 g, the indentation responses of the polished glasses samples were measured and compared. The hardness was determined using the below expression.

(4) $$H_{v}=2P/d^{2},$$

where d [m] and P [N] are the average lengths of the indent’s diagonal and indentation load, respectively. The Brewster angle (*θ_B_*) approach estimates the RI of polished glass surfaces using the formula

*RI* = tan(*θ_B_*). (5)

The measurements were carried out using a 640-nm laser.

The relative number of bridging oxygen atoms calculated per glass-forming cation ($n_{BO}$) is provided by

(6) $$n_{BO}=4-\underset{i}{\sum }M_{i}Z_{i}/(\underset{j}{\sum }F_{j}),\;\;$$

where $M_{i}\;$ is the atomic fraction, $Z_{i}$ represents the valency of the *i*th modifying cation, and $F_{j}$ is the fraction of the *j*th glass-forming cation. In the case of oxynitride glasses, the $n_{BO}$ can be calculated by replacing [O] with an equivalent anionic concentration [O*], with [O*] = [O] + 3/2[N], assuming three-fold coordinated N in the glass network. The average coordination number <*n*> was calculated according to the expression

(7) 〈n〉=4xSi−2x(Ca)+2(O−(Ca))−[Si−Ca+O+N],

where Si, Ca, O, and N are respective atomic concentrations in the glass.

## 3. Results

### 3.1. Glass Stoichiometry and Morphology

**Figure 1:**
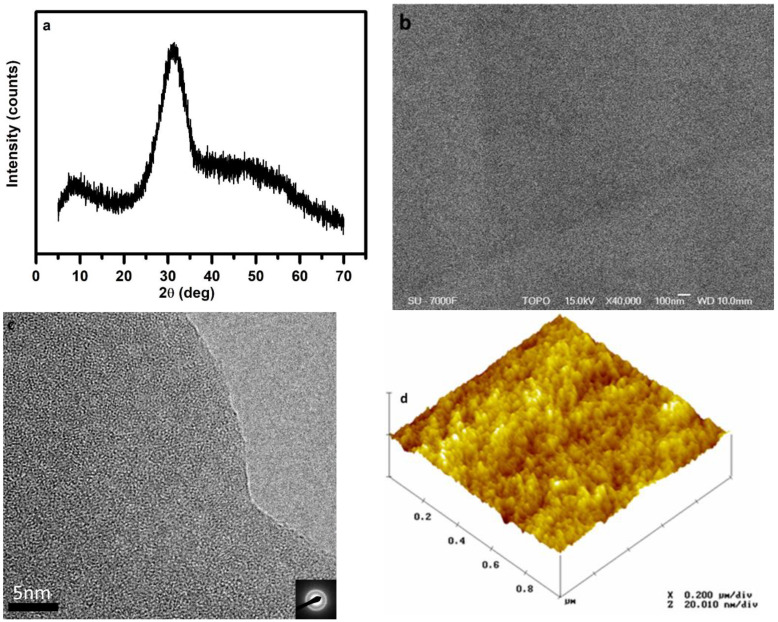
Left panel: (**a**). XRD pattern(**c**) high-resolution electron microscopy (HREM) image. Right panel: (**b**) SEM backscattered electron image and (**d**) atomic force microscopy image (AFM) of glass sample Ca_8.03_ Si_10_O_17.92_N_6.65_.

Table 1 and Table 2 summarize the compositions and a range of their analyzed property values of the Ca–Si–O–N glasses investigated in this work. All herein reported Ca–Si–O–N glasses were fabricated using fine powder of calcium hydride as a Ca source and X-ray amorphousness. A typical recorded X-ray powder pattern for pure glass is shown in Figure 1a for the glass sample Ca_8.03_ Si_10_O_17.92_N_6.65_ (cf. Table 1, sample # 8) with Ca content of 29 e/o and N content of 36 e/o, respectively. It exhibits the characteristic broad diffraction maxima of an amorphous phase. The fabricated samples in the Ca–Si–O–N matrix were mostly black or dark grey, as previously reported for oxynitride glasses in Ca-Si-(Al)-O-N systems [4,12,26,30,31,32,33]. The coloration in these glasses is related to the presence of metal silicide and a small number of Si particles. The obtained glasses comprised up to 58 e/o nitrogen and 42 e/o Ca. Some of the glasses have a higher content of glass modifier, i.e., Ca, than glass former, i.e., Si, and thus fall under the category of inverted oxynitride glasses (Ca/Si > 1). The glass composition conveys information about its average network polymerization degree through the X:Si (X= O, N) ratio, corresponding to the number of anions per tetrahedral (network forming) cation, see Table 1. X:Si ratios are found to vary between 2.22 to 2.89 and can be associated with the 3D framework and 2D sheet structure built by Q^4^ and Q^3^ tetrahedra.

The SEM backscattered electron image and high-resolution transmission electron microscopy (HRTEM) image of glass Ca_8.03_ Si_10_O_17.92_N_6.65_ (cf. Table 1, sample # 8) displayed in Figure 1b,c reveal homogenous microstructures; furthermore, selected area electron diffraction (SAED) patterns confirmed that the sample was amorphous in nature. The surface topography was investigated on the freshly fractured surfaces by atomic force microscopy (AFM). Figure 1d reveal that the glass has a smooth and uniform surface, and no metallic inclusions or other heterogeneities were detected. The SEM, HRTEM, and AFM observations confirmed that the samples are defect-free and featureless structures, characteristic of non-crystalline materials. X-ray powered diffraction, SEM, HREM, and AFM images reported in Figure 1a–d refer to the Ca_8.03_ Si_10_O_17.92_N_6.65_ sample; however, they are representative of all the studied samples.

### 3.2. Effect of Atomic Packing Density on Bridging Oxygen and Average Coordination Number

The atomic packing density $\left(C_{g}\right)$ has much less effect on bridging oxygen ($n_{BO}$) and the average coordination number 〈n〉 (see Table 1 and Table 2). Both the number of bridging oxygen and the average coordination number decrease with the increasing atomic packing density of the glass. This observation can be attributed to the fact that as the cross-linking degree of the atomic network increases, i.e., as the average coordination number or as the number of bridging oxygen atoms per silicate tetrahedron is raised, the atomic packing density decreases.

### 3.3. Effect of Atomic Packing Density on Measured Glass Density and Calculated Molar Volume

**Figure 2:**
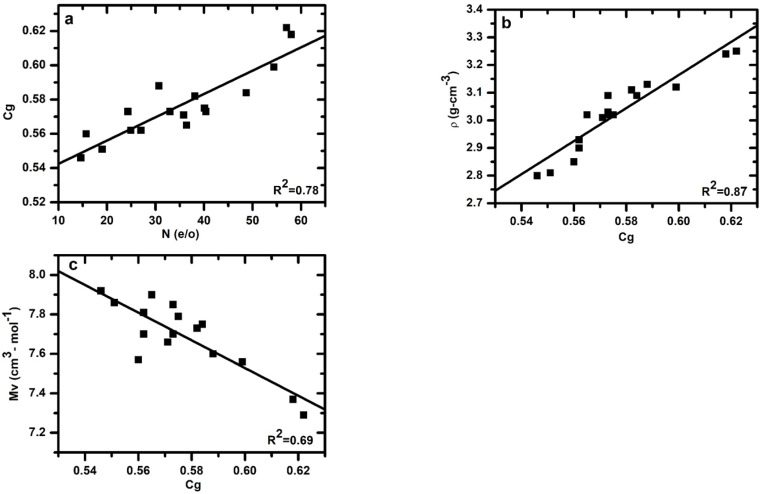
(**a**) Relation between glass atomic packing density and nitrogen content (**b**) glass density as a function of glass atomic packing density (**c**) relation between glass atomic packing density and molar volume of *Ca*–Si–O–N glasses.

The atomic packing density $\left(C_{g}\right)$ of the glass is closely related to the N content and is increased with increasing N content in the glass, as shown in Figure 2a. The $C_{g}\;$ values vary between 0.546 and 0.622. Generally, oxynitride glasses have higher values of $C_{g}$ than oxide glasses ($C_{g}$ < 0. 540) and lower values than metallic glass $C_{g}$ > 0.70 [13,27].

Measured values of the density of the oxynitride glass samples are demonstrated in Table 2. As can be observed, the density values of the samples varied from 2.80 to 3.25 g/cm^3^. The density of the glasses is plotted in Figure 2b as a function of glass atomic packing density. The density increases with increasing atomic packing density. A fit of the data to a linear dependence of density on atomic packing density yielded *ρ* =−0.42 (0.33) + 5.98(0.58) × [*C_g_*], with regression coefficient R^2^ = 0.87. The density of the Ca–Si–O–N samples exhibits dependence on *C_g_* and chemical composition.

The molar volume value spans 7.29 cm^3^/mol to 7.92 cm^3^/mol. The molar volume decreases with increasing the atomic packing density of the glass and increases with increasing the modifier content, in agreement with previously reported data on other oxynitride systems. Mv *=* 11.73 (0.67) – 7.02(1.15) × [*C_g_*], with regression coefficient R^2^ = 0.69. The relationship between the compactness and molar volume is shown in Figure 2c.

### 3.4. Effect of Atomic Packing Density on Glass Transition and Crystallization Temperatures

The glass transition temperature $\;\left(T_{g}\right)$ of the oxynitride glass samples depends on the N content and structural parameters. The $T_{g}$ is expected to increase with the N content, M-O bond strengths, glass cross-linking, and *C_g_*. The $T_{g}\;$ and $T_{c}$ for glasses in the Ca–Si–O–N system are summarized in Table 2, and range from 800 °C to 1050 °C, and from 930 °C to 1220 °C for $T_{g}$, and $T_{c},$ respectively. The average temperature difference between $T_{g}$ and *T_c_* is approximately 130 °C.

**Figure 3:**
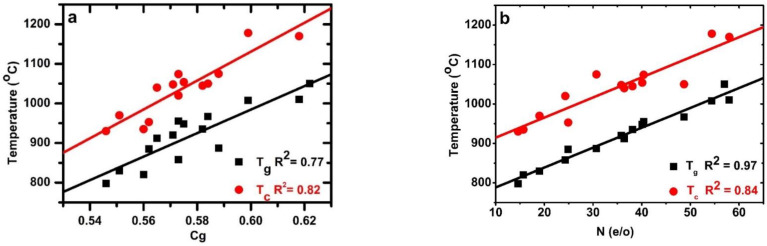
(**a**) Glass transition and crystallization temperatures as a function of glass atomic packing density (**b**) glass transition and crystallization temperatures as a function of nitrogen content.

Figure 3a show that $T_{g}$ increases concomitantly with the Cg of the glass since as the addition of N content increases the $C_{g}$ of the glass, the $T_{g}$ is expected to increase. The linear dependency between $T_{g}$ and $T_{c}$ of the samples due to the $C_{g}$ yielded $T_{g}$ = −800(250) + 2971(438) × [*C_g_*] with regression coefficient R^2^ = 0.77 and $T_{c}$ = −1058(273) + 3650(480) × [*C_g_*] with R^2^ = 0.82. The dependency of $T_{g}$ on N content is more obvious, as shown in Figure 3b. Fitting the data to a linear dependence of $T_{g}$ on N content yielded $T_{g}$= 738 (9) + 5 (25) × [N], with R^2^ = 97 and $T_{c}$ *=* 863 (23) + 5 (62) ×[N] with R^2^ = 0.84.

### 3.5. Effect of Atomic Packing Density on Hardness

**Figure 4:**
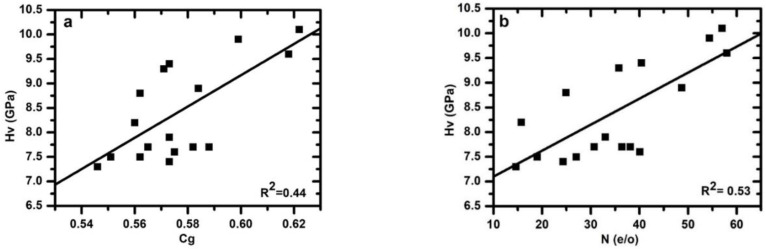
(**a**) Microhardness as a function of glass atomic packing density (**b**) microhardness as a function of nitrogen content (in e/o).

Permanent deformation is characterized by the Vickers hardness $\left(H_{v}\right)$. In Figure 4a,b the mean hardness values of 10 tests are plotted against their corresponding atomic packing density values and N content, respectively, for the Ca–Si–O–N glasses analyzed in this study. The hardness values range from 7.35 to 10.12 GPa and slightly increase with the atomic packing density. A fit of the obtained data to a linear dependence of hardness on compactness yielded $H_{v}$ =−10 (5) + 32 (8) × [*C_g_*], with R^2^ = 0.44 and the dependence of hardness on N yielded $H_{v}$ = 6.52 (0.46) + 0.05(0.01) × [N], with R^2^ = 0.53.

### 3.6. Effect of Atomic Packing Density on Refractive Index

**Figure 5:**
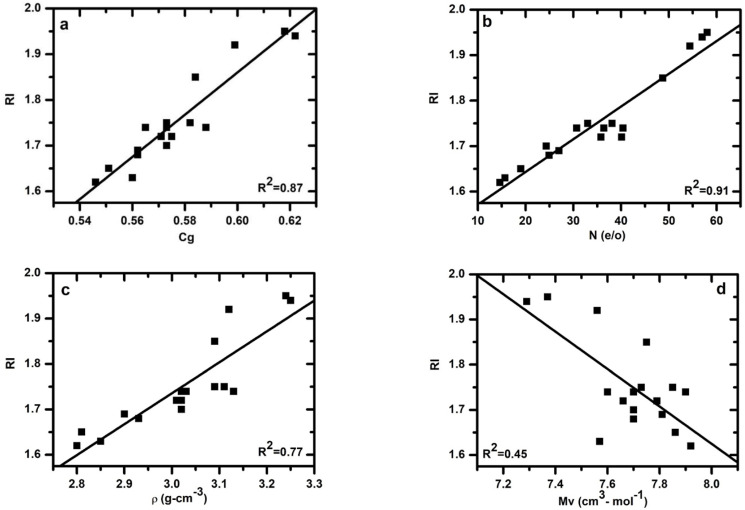
Left panel: (**a**) Glass refractive index as a function of atomic packing density (**c**) relation between refractive index and density. Right panel: (**b**) glass refractive index as a function of nitrogen content (in e/o) (**d**) relation between refractive index and molar volume for Ca–Si–O–N glasses.

The *RI* depends primarily on the polarizability of the various constituent ions of materials and the bonds between atoms. Figure 5a show that there is a substantial increase in *RI* with the increasing atomic packing density of the glasses. Figure 5b show the relationship between the *RI* and N content. A fit of the obtained data to a linear dependence of *RI* on $C_{g}$ yielded RI = -0.92 (0.25) + 4.62(0.43) × [*C_g_*], with R^2^ = 0.87 and the dependence of RI on N content yielded *RI* = 1.49 (0.02) + 0.0072 (0.0005) × [N], with R^2^ = 0.91, indicating that the $C_{g}$ and N content have a similar effect on the *RI*. The strong relationship between the density and *RI* of Ca–Si–O–N is expected when considering the result of Figure 5c and their corresponding correlation coefficient with R^2^ = 0.77. Indeed, the refractive index of an oxide-based glass is normally governed by both the atom/ion polarizabilities and the packing density of the constituent atom.

## 4. Discussion

For most glasses except oxynitride ones, the atomic packing density decreases as the cross-linking degree of the atomic network increases. This is because as the cross-link develops, the glass network becomes less compliant, and the atomic topology is governed more and more by the glass former-oxygen skeleton [34,35,36,37]. For instance, $C_{g}$ is typical of the order of 0.45 for amorphous silica ($n_{BO}$ = 4 and 〈n〉 = 2.67), 0.5 for soda–lime–silica glasses (typical window glasses, $n_{BO}\;$ is between 2 and 3), and above 0.7 for most bulk metallic glasses. In this regard, silicon oxynitride glasses behave anomalously since they exhibit $C_{g}$ values typically larger than 0.55, although N is supposed to significantly increase the connectivity [27,38]. A striking feature is that $C_{g}$ increases with the N content in spite of the fact that N is three-fold coordinated. For instance, in the Ca–Si–O–N system [13], the atomic packing density, as well as glass density, increases with the N content, whereas in the Ca–Si–O glass system [39], it was found that the atomic packing density decreases as the Ca content decreases and the number of bridging oxygen increases. Furthermore, glasses in the Ca–Si–O–N system containing SiO_3_N groups might accommodate more Ca in sites than the equivalent oxide samples without generating non-bridging oxygen species.

Our observed monotonic increase in density and decrease in the molar volume against the glass atomic packing density mirrors the fact that C_g_ induces contraction in the glass network. Numerous reports on oxynitride systems, e.g., AE–Si–(Al)–O–N [4,14,15,28,30,40,41,42] and RE–Si–(Al)–O–N [9,10,23,25,43,44,45,46,47,48,49], show that the density is more dependent on the modifier content and concentration than the N content. A linear regression fit on both fractional glass compactness and N content yielded *ρ =* 0.24 (0.66) + 4.7 (1.3) × [*C_g_*] + 0.01(0.01) × [N] with R^2^ = 0.87. The data thus indicate that ρ primarily depends on $C_{g}$. Conversely, the Mv, which is inversely proportional to density, decreases non-linearly with increasing $C_{g}$ of the Ca–Si–O–N glasses. The Mv is also affected by modifier cations [7,50,51,52]. In earlier reports on N-rich oxynitride glasses in the Ca–Si–O–N [12,28], Sr–Si–O–N [14], and Ba–Si–O–N [15] systems, it was found that increasing $C_{g}$ reduces the Mv and increasing content of modifier cation increases it.

The glass transition temperature $\left(T_{g}\right)$ depends on the chemical composition, atomic packing density, and strength of the cation–anion bonds. Since an increase in N content increases the atomic packing density of glass, the cross-linking of the glass network increases, so the $T_{g}$ is expected to increase alongside the $C_{g}$. The $T_{g}$ and $T_{c}\;$ increase with increasing nitrogen content due to an increase in the coordination of the anion and a stronger covalent bond between Si–N versus Si–O. The increase in $T_{g}$ with the N content is consistent with alkaline–earth containing oxynitride glasses [4,12,15,26,30,41,50,53,54,55].The improvement in $T_{g}\;$ originates from the increase of atomic network cross-linking, due to the three-fold coordinated nitrogen and the atomic packing density, despite nitrogen being lighter than oxygen and the Si–O bond being stronger than the Si–N bond. $T_{g}$ is also affected by CFS, and for high CFS, the effective force attracting anions increases; consequently, the glass network becomes more compact, which results in $T_{g}$ increase. For example, the glasses in the Mg–Si–O–N system have higher $T_{g}$ values than in Ca/Sr/Ba–Si–O–N [26] glasses. The regression analysis yielded $T_{g}$ = 670 (200) + 5 (1) × [N] + 160 (370) × [*C_g_*] with R^2^ = 0.97. The present data thus indicate that $T_{g}$ is more dictated by N content as compared to the $C_{g}$ of the glass.

The microhardness of the oxynitride glass and the silicate-based oxide glass is dictated by many factors such as the bond strength, $C_{g},$ and the polymerization/topology of the network of interconnected SiO_4_ and AlO_4_ tetrahedra [15,20,24,25,27,56,57,58,59]. Generally, microhardness diminishes with increasing alkali or alkaline–earth ion content in both oxide and oxynitride glass networks, which cause depolymerization of the network by converting bridging oxygen ($n_{BO}$) into non-bridging oxygen ($n_{NBO}$) species [11,26,60]. N is well known in oxynitride glass systems to increase hardness. Hardness has frequently been related to the CFS of the modifier elements and varies substantially with the type of modifier; e.g., Mg- and Ca-containing glasses exhibit higher hardness values than corresponding Sr- and Ba-containing glasses [26,30]. Furthermore, hardness recurrently increases with the increase in CFS. Generally, the effects of replacing N for O and modifier cation replacement on hardness are independent of each other and additive rather than synergistic. The present data in the Ca–Si–O–N system do not show a clear trend. A fit of the data to linear dependence of hardness on nitrogen and atomic packing density yielded $H_{v}$ = 2.8 (9.5) + 0.05 (0.03) × [N] + 7 (18) × [*C_g_*] with R^2^ = 0.50. The regression analysis shows that the hardness is more dependent on the N content as compared to the $C_{g}$. It is also possible that different atomic packing density values might correspond to the same microhardness because different mechanisms produce the same permanent deformation.

The refractive index (*RI*) is found to be increased with both increasing N content and changed with the amount of glass modifier. In addition, it was observed that the amount of glass modifier depends on the atomic number of the modifier element. N-rich glasses in the systems AE–Si–O–N with AE = Mg, Ca, Sr, and Ba also show that the *RI* depends on the N content and the amount and type of modifiers [26]. For the present Ca–Si–O–N glasses, the *RI* varies between 1.62 and 1.94 and increases linearly with the atomic packing density of the glass. The *RI* of glass is dependent on the polarizability and molar volume of the glass [61,62]. The addition of cation modifiers in the glasses network leads to a more open structure. Moreover, the addition of cation modifiers causes the glass structure to expand to accommodate the cations at interstitial sites. Therefore, this increase in molar volume, having a considerable effect compared to any polarizability effect, leads to a decrease in the *RI*, as shown in Figure 5d. The addition of N in the glass network increases structural polymerization. This creates a more rigid network and causes a decrease in the molar volume leading to a higher *RI*. From the present data, it is clear that *RI* decreases with increasing Mv. In previous studies of Sr–Si–O–N [14] and Ba–Si–O–N [15] systems, it was found that modifiers are slightly more effective on the *RI* than the N content. The Ba-containing glasses show high RI values compared to the Sr- and Ca-containing glasses. A fit of the data to a linear dependence of *RI* on N and atomic packing density yielded *RI* = 0.40 (0.33) + 0.0044 (0.0009) × [N] + 2.08 (0.63) × [*C_g_*] with R^2^ = 0.95. The present data demonstrate that *RI* is correlated to both the N content and the $C_{g}$ of the glass.

## 5. Conclusions

Physical and optical properties such as glass transition temperature, density, hardness, molar volume, crystallization temperature, and refractive index were studied as a function of atomic packing density for 17 calcium silicon oxynitride glasses with different Ca (15 to 42 e/o) and N (15 to 58 e/o) contents. Glasses in the Ca–Si–O–N matrix with a high atomic packing density exhibit relatively strong interatomic interactions and sturdily affect some properties. The number of bridging oxygen and average coordination number manifested non-linear relationships with the atomic packing density. The density and refractive index are found to increase predominantly with the atomic packing density but also with the Ca content. Indeed, the refractive index of an oxynitride-based glass is normally governed by both the atom/ion polarizabilities and the packing density of the constituent atom. The glass transition and crystallization temperatures and molar volume increase and decrease, respectively, with increasing the atomic packing density. Unexpectedly no strong correlation between the microhardness and atomic packing density of the Ca–Si–O–N glasses was observed, indicating that the atomic packing density is not solely controlled by the underlying deformation mechanism. The regression analysis shows that the microhardness is more dominated by the N content compared to the atomic packing density.

## Figures and Tables

**Table 1:** Data for prepared Ca–Si–O–N glasses, determined glass composition, Ca content in e/o, Si content in e/o, O content in e/o, N content in e/o, X:Si ratio = [O,N]/[Si], number of bridging oxygen ($n_{BO}$), and average coordination number <n>.

Glass ID	Glass Composition	*Ca/* *e/o*	*Si/* *e/o*	*O/* *e/o*	*N/* *e/o*	*X:Si*	$n_{BO}$	〈n〉
1	Ca_3.41_Si_10_O_19.75_N_2.45_	14.6	85.4	84.3	15.7	2.22	3.709	2.543
2	Ca_5.29_Si_10_O_21.61_N_2.46_	20.9	79.1	85.4	14.6	2.41	2.582	2.413
3	Ca_4.90_Si_10_O_20.17_N_3.15_	19.7	80.3	81.0	19.0	2.33	3.606	2.470
4	Ca_6.52_Si_10_O_20.02_N_4.43_	24.6	75.4	75.1	24.9	2.45	3.511	2.408
5	Ca_9.3_Si_10_O_22.18_N_4.75_	31.8	68.2	75.7	24.3	2.69	3.365	2.223
6	Ca_6.67_Si_10_O_19.47_N_4.8_	25.0	75.0	73.0	27.0	2.43	3.500	2.415
7	Ca_11.81_Si_10_O_22.06_N_6.50_	37.1	62.9	69.3	30.7	2.86	3.258	2.108
8	Ca_8.03_Si_10_O_17.92_N_6.65_	28.7	71.3	64.2	35.8	2.46	3.424	2.399
10	Ca_14.65_Si_10_O_23.12_N_7.69_	42.0	58.0	67.0	33.0	3.08	3.155	1.935
9	Ca_9.14_Si_10_O_17.37_N_7.84_	31.4	68.6	59.6	40.4	2.52	3.373	2.367
11	Ca_12.90_Si_10_O_20.93_N_7.98_	39.2	60.8	63.6	36.4	2.89	3.216	2.084
12	Ca_9.94_Si_10_O_17.73_N_8.14_	33.2	66.8	59.9	40.1	2.59	3.336	2.319
13	Ca_12.91_Si_10_O_20.37_N_8.36_	39.3	60.7	61.9	38.1	2.87	3.215	2.098
14	Ca_11.77_Si_10_O_16.30_N_10.31_	37.1	62.9	51.3	48.7	2.66	3.259	2.273
15	Ca_9.74_Si_10_O_13.57_N_10.78_	32.8	67.2	45.6	54.4	2.44	3.345	2.459
16	Ca_10.07_Si_10_O_12.92_N_11.40_	33.4	66.6	43.0	57.0	2.43	3.329	2.464
17	Ca_11.04_Si_10_O_13.21_N_11.89_	35.6	64.4	42.0	58.0	2.51	3.289	2.408

**Table 2:** Physical properties of Ca–Si–O–N glasses: glass designation, density (*ρ*), molar volume (Mv), glass compactness ($C_{g}$), glass transition temperature ($T_{g}$), crystallization temperature ($T_{c}$), Vickers hardness ($H_{v}$), and refractive index (*RI*). Numbers in parentheses are estimated standard deviations.

Glass ID	$C_{g}$	*ρ/* gm·cm^−3^	Mv/ cm^3^·mol^−1^	$T_{g}/$ °C	$T_{c}/$ °C	$H_{v}/$ GPa	*RI*
1	0.560	2.85	7.57	820	935	8.2(2)	1.63
2	0.546	2.80	7.92	798	930	7.3(5)	1.62
3	0.551	2.81	7.86	830	970	7.5(4)	1.65
4	0.562	2.93	7.70	885	953	8.8(83)	1.68
5	0.573	3.02	7.70	858	1020	7.4(5)	1.70
6	0.562	2.90	7.81	-	-	7.5(4)	1.69
7	0.588	3.13	7.60	887	1075	7.7(3)	1.74
8	0.571	3.01	7.66	920	1048	9.3(5)	1.72
9	0.573	3.09	7.85	-	-	7.9(3)	1.75
10	0.573	3.03	7.70	955	1074	9.4(8)	1.74
11	0.565	3.02	7.90	912	1040	7.7(5)	1.74
12	0.575	3.02	7.79	948	1054	7.6(3)	1.72
13	0.582	3.11	7.73	935	1045	7.7(4)	1.75
14	0.584	3.09	7.75	967	1050	8.9(3)	1.85
15	0.599	3.12	7.56	1008	1178	9.9(5)	1.92
16	0.622	3.25	7.29	1050	-	10.1(2)	1.94
17	0.618	3.24	7.37	1010	1170	9.6(2)	1.95
Experimental uncertainties	±0.001	±0.02	±0.01	±5	±5		±0.02

## Data Availability

Not applicable.
